# Tobacco use among non-elderly adults with and without criminal justice involvement in the past year: United States, 2008–2016

**DOI:** 10.1186/s13722-019-0131-y

**Published:** 2019-01-11

**Authors:** Tyler N. A. Winkelman, Katherine Diaz Vickery, Andrew M. Busch

**Affiliations:** 1 Department of Medicine, Hennepin Healthcare, Hennepin County Medical Center, 701 Park Ave, S2.309, Minneapolis, MN 55415 USA; 2 Hennepin Healthcare Research Institute, 701 Park Ave, Suite PP7.700, Minneapolis, MN 55415 USA

**Keywords:** Tobacco use, Smoking, Cigarettes, Criminal justice, Disparities

## Abstract

**Background:**

Tobacco use remains the leading cause of preventable disease and death in the United States and is concentrated among disadvantaged populations, including individuals with a history of criminal justice involvement. However, tobacco use among individuals with a history of criminal justice involvement has been understudied in the United States, and data are needed to inform policy and practice.

**Methods:**

We used data from the 2008–2016 National Survey on Drug Use and Health (unweighted N = 330,130) to examine trends in tobacco use, categories of tobacco use, characteristics of cigarette use, and health care utilization and tobacco use screening among individuals (aged 18–64) with and without a history of criminal justice involvement in the past year. We used multiple logistic and Poisson regression models with predictive margins to provide adjusted prevalence estimates.

**Results:**

The weighted sample in each year was, on average, representative of 8,693,171 individuals with a history of criminal justice involvement in the past year and 182,817,228 individuals with no history of criminal justice involvement in the past year. Tobacco use was significantly more common among individuals with a history of criminal justice involvement compared with individuals with no criminal justice involvement, and disparities increased over time (Difference in adjusted relative differences: − 10.2% [95% CI − 17.7 to − 2.7]). In 2016, tobacco use prevalence was more than two times higher among individuals with a history of criminal justice involvement (62.9% [95% CI 59.9–66.0] vs. 27.6% [95% CI 26.9–28.3]). Individuals with a history of criminal justice involvement who smoked reported a significantly earlier age of cigarette initiation, more cigarettes used per day, and higher levels of nicotine dependence and chronic obstructive pulmonary disease. Individuals with a history of criminal justice involvement were less likely to report an outpatient medical visit in the past year and, among those reporting an outpatient medical visit, were less likely to be asked about tobacco use, but paradoxically, more likely to report being advised to quit.

**Conclusions:**

Novel programs and tobacco control policies are needed to address persistently high rates of tobacco use and reduce cardiovascular morbidity and mortality among individuals with a history of criminal justice involvement.

## Background

Tobacco use remains the leading cause of preventable disease and death in the United States [1]. Cigarette smoking, the most common form of tobacco use, is responsible for over 450,000 deaths and $300 million in economic costs every year [2]. While smoking rates among the general US population have declined substantially over the past several decades, decreases have been disproportionately concentrated among higher-income groups [1, 3]. Therefore, smoking is now highly concentrated among disadvantaged populations, including individuals involved in the criminal justice system (i.e., individuals who have been incarcerated in jail or prison, on probation/parole, or arrested) [4, 5]. High levels of smoking among individuals in prisons contribute to excess age-adjusted mortality and years of potential life lost in this population [6, 7].

Tobacco use among individuals involved in the criminal justice system represents a critical public health concern because over 6.5 million individuals are under correctional control in the United States on any given day [7–12]. Nonetheless, the issue has received limited research attention and epidemiological data are sparse. Most data are regional or were collected more than a decade ago [9, 10]. Few studies encompass the largest population of individuals involved in the criminal justice system, those who are not incarcerated but still under correctional control (i.e., probation, parole, or arrest) [7, 13–15]. The most recent available estimates of individuals with a history of involvement in the US criminal justice system are from 2006, when the Centers for Disease Control and Prevention reported 56.2% of individuals who spent at least 1 day “on the streets, in a shelter, or in a jail or prison” were current smokers. Further, national data regarding other types of tobacco use among this population and differences between individuals with and without a history of criminal justice involvement who use tobacco are not available. Such data could inform policy and practice to reduce tobacco-related morbidity among individuals with a history of involvement in the criminal justice system.

We used the most recently available US data to examine trends in tobacco use among individuals with criminal justice involvement in the past year compared with the general population from 2008 to 2016. Among individuals with and without a history of criminal justice involvement, we also compared categories of tobacco use (i.e., cigarettes only, cigars only, smokeless tobacco only, or combination tobacco use), characteristics of cigarette use among individuals who smoke, and health care utilization and tobacco use screening among those with any tobacco use.

## Methods

### Data source and study population

We used data from the 2008–2016 National Survey on Drug Use and Health (NSDUH), the primary nationally representative source of estimates of drug use and mental illness of the US population aged 12 and older. The NSDUH is a cross-sectional, household survey that uses a combination of computer-assisted personal interviewing, with an interviewer present, and audio computer-assisted self-interviewing to support confidentiality and privacy for sensitive questions [16]. Over 55,000 individuals are surveyed annually, including some individuals with no permanent housing (e.g., residence in a homeless shelter), but not those in jail or prison or who are homeless and do not live in a recognized shelter for homeless individuals. Weighted interview response rates are generally around 70% [16, 17].

We limited our study population to non-elderly adults aged 18–64, because over 97% of individuals involved in the criminal justice system are in this age range [18]. Our primary independent variable of interest was history of criminal justice involvement in the past year. We identified an individual as having been involved in the criminal justice system if they reported an arrest or time on probation or parole in the past 12 months. Less than one percent of our sample were missing criminal justice history data.

### Tobacco use trends

Our primary outcome variable was any tobacco use in the past month. An individual was determined to have used tobacco in the past month if they reported any use of cigarettes, cigars (i.e., “big cigars, cigarillos, and even little cigars that look like cigarettes”), smokeless tobacco (i.e., “snuff, dip, chewing tobacco, or snus”), or a tobacco pipe in the past month. We used imputation-revised frequencies provided by the NSDUH, and therefore, all observations had complete past month tobacco use data. Generally, less than 0.5% of observations had missing data requiring imputation for tobacco-related variables.

Mutually exclusive categories of tobacco use, including cigarette use only, cigar use only, smokeless tobacco only, and combination tobacco use were explored. Variables were defined by self-report within each tobacco category. Combination tobacco use was defined as use of two or more categories of tobacco use.

### Characteristics of cigarette use and co-occurring health conditions

Because cigarette use represents the majority of tobacco use and tobacco-related morbidity in the United States [2], we compared characteristics of cigarette use among individuals with and without criminal justice involvement who reported any cigarette use in the past month. Characteristics of cigarette use included age at first cigarette use, cigarettes per day, and nicotine dependence. Cigarettes per day was measured in ranges (e.g., 6–15 cigarettes per day, 26–35 cigarettes per day). We used the midpoint of each range and top-coded the highest category (i.e., more than 35 cigarettes) at 50 cigarettes. The NSDUH measures nicotine dependence using both the Fagerstrom Test of Nicotine Dependence [19] and the Nicotine Dependence Syndrome Scale [20]. Respondents who meet criteria for dependence on either scale were considered to be nicotine dependent.

In addition, we assessed co-occurring chronic conditions, substance use disorders, and serious mental illness among individuals with and without criminal justice involvement in the past year who reported cigarette use in the past month. We examined chronic obstructive pulmonary disease (COPD) and heart disease because they are long-term health consequences of cigarette use [21]. Individuals were asked, in the 2015 and 2016 NSDUH, whether they had ever been told by a doctor or health care professional they had COPD or a heart condition. These same questions were not available in earlier years of the survey. In addition, we examined the prevalence of alcohol use disorders, illicit drug use in the past year (excluding marijuana), marijuana use in the past year, and serious mental illness, which are known to be more prevalent among individuals who smoke [22, 23].

### Health care utilization and tobacco use screening among individuals with tobacco use in the past month

Finally, we measured outpatient visits (1 or more vs. none) among individuals who reported any category of tobacco use in the past month. Among those with at least one outpatient visit, we assessed whether they were asked about or advised to quit their tobacco use during medical visits. The U.S. Preventive Services Task Force recommends all adults be asked about tobacco use and, if using tobacco, advised to quit [24].

### Sociodemographic characteristics

We assessed age, race/ethnicity, and gender of our study population. We controlled for sociodemographic differences between individuals with and without criminal justice involvement in the past year in all analyses.

### Statistical analysis

We estimated weighted frequencies of sociodemographic characteristics and used Pearson’s Chi squared test for statistical comparisons.

We used multiple logistic regression and predictive margins to examine the adjusted prevalence of tobacco use in the past month over each study year among individuals with and without criminal justice involvement in the past year.

We used similar regression models to compare tobacco use categories among individuals with tobacco use in the past month, characteristics of cigarette use and health conditions among individuals with cigarette use in the past month, and health care utilization and tobacco use screening among individuals with any category of tobacco use in the past month. These models included data from the 2015 and 2016 NSDUH only. We converted adjusted odds ratios to adjusted prevalence using predictive margins. We used Poisson models with robust standard errors, rather than logistic regression, to measure age of first cigarette use and average cigarettes per day. Key moderators between advice to quit tobacco use and criminal justice involvement were assessed by sequentially incorporating sociodemographic characteristics.

All analyses accounted for the complex survey design of NSDUH by using person-level analysis weights, which allowed for nationally representative inferences. Each weight used in this analysis is the result of 16 weight components that account for selection probability, nonresponse, coverage, or extreme weights. The sum of the person-level analysis weights represents an estimate of the individuals in a given population; for purposes of this study, non-elderly adults in the United States. It is standard practice to report weighted, nationally representative estimates from these data [25]. Additional information regarding generation of survey weights in NSDUH is described in detail elsewhere [16]. We used Stata MP 15.1 for Mac (*StataCorp,* College Station, TX) and considered two-sided *P* < .05 to be statistically significant. We followed the STROBE reporting guidelines for cross-sectional studies (e.g., clear variable specification, description of statistical analysis, reporting 95% confidence intervals) [26].

## Results

### Study population

Our weighted sample in each year was, on average, representative of 8,693,171 individuals with a history of criminal justice involvement in the past year (N = 21,466) and 182,817,228 individuals with no history of criminal justice involvement in the past year (N = 308,664; total unweighted N = 330,130; respondents in each year: 2008—35,318; 2009—35,405; 2010—35,588; 2011—36,454; 2012—35,242; 2013—34,826; 2014—37,831; 2015—39,725; 2016—38,741). Among individuals who reported criminal justice involvement in the past year, 70.9% reported an arrest in the past year, 54.8% reported probation in the past year, and 17.8% reported parole in the past year. Individuals with a history of criminal justice involvement in the past year were more likely to be male, African-American, Hispanic, and younger (Table 1).

**Table 1:** Characteristics of study population by history of criminal justice involvement, United States 2008–2016

Characteristic	Weighted% (95% CI)		*P* value
	Past year criminal justice involvement (N = 21,466)	No past year criminal justice involvement (N = 308,664)	
Male	71.5 (70.6–72.4)	48.0 (47.7–48.2)	< .001
Race/ethnicity			< .001
White, non-Hispanic	55.1 (53.9–56.2)	64.3 (63.9–64.7)	
African-American, non-Hispanic	21.0 (20.1–21.9)	11.9 (11.6–12.1)	
Hispanic	19.0 (18.0–20.0)	16.1 (15.8–16.4)	
Other	5.0 (4.6–5.5)	7.8 (7.5–8.0)	
Age			< .001
18–25	32.8 (31.9–33.7)	17.1 (16.9–17.4)	
26–34	26.8 (25.5–28.0)	18.9 (18.7–19.2)	
35–49	26.9 (25.9–28.0)	32.3 (32.0–32.5)	
50–64	13.5 (12.4–14.7)	31.7 (31.3–32.1)	

### Tobacco use trends

**Fig. 1:**
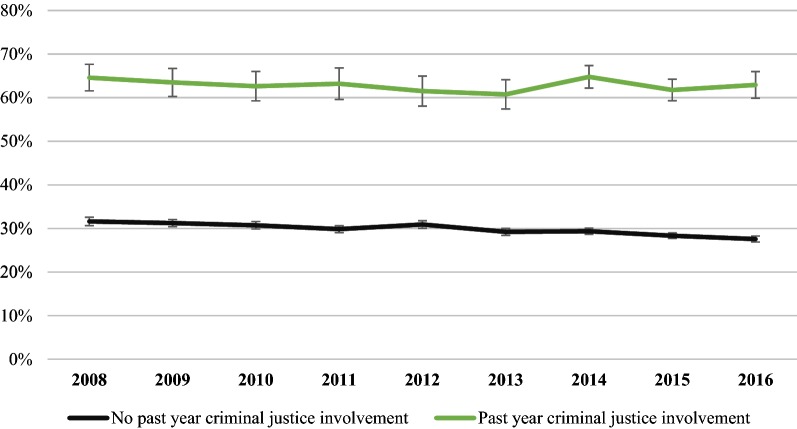
Past month tobacco use by history of criminal justice involvement, United States 2008–2016. Estimates are adjusted for age, race/ethnicity, and sex

Individuals with a history of criminal justice involvement in the past year had persistently higher levels of tobacco use in the past month compared to individuals with no history of criminal justice involvement (Fig. 1). Among individuals with criminal justice involvement in the past year, 64.6% (95% CI 61.5–67.6) reported tobacco use in the past month in 2008 and 62.9% (95% CI 59.9–66.0) reported tobacco use in the past month in 2016, a difference that was not statistically significant (Adjusted relative difference [ARD], − 2.6% [95% CI − 9.1–3.9]). In comparison, tobacco use declined significantly among individuals with no criminal justice involvement in the past year. Tobacco use prevalence among the general population declined from 31.6% (95% CI 30.6–32.6) in 2008 to 27.6% (95% CI 26.9–28.3) in 2016 (ARD, − 12.8% [95% CI − 16.2, − 9.4]). The adjusted relative difference was significantly lower among individuals with no criminal justice involvement in the past year compared with individuals with past year criminal justice involvement (Difference in ARD, − 10.2% [95% CI − 17.7, − 2.7]).

**Fig. 2:**
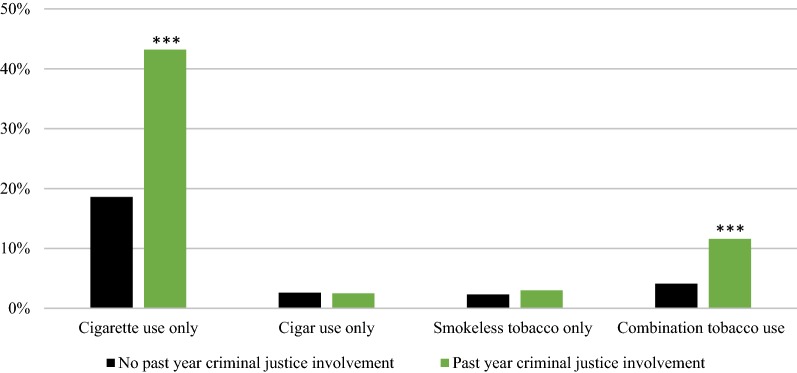
Categories of tobacco use by history of criminal justice involvement, United States 2015–2016. Estimates are adjusted for age, race/ethnicity, and sex. ***P < .001 compared to “No past year criminal justice involvement”

Higher prevalence of tobacco use in the past month among individuals with criminal justice involvement in the past year compared to those with no criminal justice involvement was due to significantly higher use of cigarettes only (43.2% [95% CI 41.0–45.5] vs. 18.6% [95% CI 18.2–19.0]) and combination tobacco products (11.6% [95% CI 10.4–12.7] vs. 4.1% [95% CI 3.9–4.2]; Fig. 2). Use of cigars only (2.5% [95% CI 1.8–3.2] vs. 2.6% [95% CI 2.5–2.8]) and smokeless tobacco only (3.0% [95% CI 2.2–3.7] vs. 2.3% [95% CI 2.2–2.5]) were statistically similar in both groups.

Among the population of individuals who used tobacco in the past month, most reported any cigarette use (i.e., cigarette use only or combination tobacco use that included cigarette use). However, among individuals who reported tobacco use, cigarette use was significantly higher for individuals with criminal justice involvement compared with individuals with no criminal justice involvement (89.7% [95% CI 87.8–91.5] vs. 80.4% [95% CI 79.6–81.2]).

### Characteristics of cigarette use and health conditions among individuals with past month cigarette use

Among individuals who reported cigarette use in the past month, those with criminal justice involvement in the past year reported an earlier age of first cigarette use, use of more cigarettes per day, and higher levels of nicotine dependence compared with those with no criminal justice involvement (Table 2).

**Table 2:** Characteristics of cigarette use and health conditions among individuals with past month cigarette use by history of criminal justice involvement, United States 2015–2016

Cigarette use characteristic	Weighted% (95% CI)^a^		*P* value
	Past year criminal justice involvement (N = 2427)	No past year criminal justice involvement (N = 17,571)	
Age of first cigarette use	14.8 (14.5–15.2)	15.8 (15.7–15.9)	< .001
Cigarettes per day	13.1 (12.5–13.8)	10.5 (10.3–10.7)	< .001
Nicotine dependence	71.6 (69.2–74.1)	54.6 (53.6–55.6)	< .001
Health condition			
COPD	9.0 (7.0–11.0)	5.7 (5.2–6.3)	.002
Heart condition	7.4 (5.4–9.5)	6.5 (5.9–7.2)	.41
Alcohol use disorder	23.7 (21.1–26.4)	12.1 (11.5–12.7)	< .001
Illicit drug use, other than marijuana^b^	37.6 (34.6–40.5)	18.5 (17.7–19.3)	< .001
Marijuana use^b^	40.5 (37.8–43.2)	30.7 (29.9–31.5)	< .001
Serious mental illness	11.9 (10.3–13.5)	7.0 (6.6–7.5)	< .001

^a^Estimates are adjusted for age, race/ethnicity, and gender

^b^In the past year

COPD was 56.7% (95% CI 21.0–92.4) higher among individuals with criminal justice involvement in the past year. Individuals with and without criminal justice involvement in the past year reported similar levels of heart condition diagnoses. Individuals with criminal justice involvement in the past year and cigarette use in the past month were significantly more likely to have a co-occurring alcohol use disorder (23.7% vs. 12.1%; *P *< .001), used an illicit drug (37.6% vs. 18.5%; *P *< .001) or marijuana (40.5% vs. 30.7%; *P *< .001) in the past year, or serious mental illness (11.9% vs. 7.0%; *P *< .001) compared with individuals with no criminal justice involvement who reported past month cigarette use.

### Health care utilization and tobacco use screening among individuals with tobacco use in the past month

Among individuals who reported tobacco use in the past month, those with criminal justice involvement in the past year were less likely to report any outpatient visit in the past year compared to those with no criminal justice involvement (Table 3). Further, among individuals with both tobacco use in the past month and any outpatient visit, those who reported criminal justice involvement in the past year were less likely to have been asked by a health professional about their tobacco use. In unadjusted analyses, individuals with criminal justice involvement were not significantly more or less likely to be advised to quit tobacco use compared to the general population (57.5% vs. 58.1%; *P* = .75). However, after adjustment, those with criminal justice involvement were more likely to be advised to quit smoking. The association of advice to quit tobacco use and history of criminal justice involvement was not significant until age was incorporated into the multiple logistic regression model; individuals 18–25 were significantly less likely to receive advice and significantly more likely to have a history of incarceration compared to older individuals. Among those who were asked about their smoking, individuals with criminal justice involvement were significantly more likely to report being advised to quit compared with individuals with no criminal justice involvement (71.4% vs. 64.7%; *P *= .001).

**Table 3:** Health care utilization and tobacco use screening among individuals with tobacco use in the past month by history of criminal justice involvement, United States 2015–2016

Characteristic	Weighted% (95% CI)^a^		*P* value
	Past year criminal justice involvement (N = 2708)	No past year criminal justice involvement (N = 21,693)	
Any outpatient visit in past year	71.8 (69.6–73.9)	74.3 (73.4–75.2)	.02
Asked by a health professional about tobacco use^b^	82.4 (89.8–85.0)	85.9 (85.1–86.6)	.01
Advised to stop using tobacco by a health professional^b^	62.4 (58.8–66.0)	57.6 (56.5–58.8)	.02

^a^Estimates are adjusted for age, race/ethnicity, and gender

^b^Among individuals with 1 or more outpatient visit in the past year

## Discussion

Among a nationally representative sample of non-elderly adults, tobacco use was more than twice as common among individuals with criminal justice involvement in the past year compared to those with no criminal justice involvement. Disparities between these two groups grew over time. Tobacco use prevalence declined 12.8% among individuals with no criminal justice involvement—five times larger than the change among individuals with criminal justice involvement in the past year (− 2.6%). Current approaches to tobacco use reduction, including public health efforts [27–29] and interventions within health care settings [30, 31], have not had a measurable impact among individuals with a history of criminal justice involvement on a population level. New approaches are needed to reduce tobacco use disparities among individuals involved in the criminal justice system.

Nearly 90% of individuals with a history of criminal justice involvement who used tobacco in the past month reported cigarette use. Not only was cigarette use more common, but the intensity of use was substantially higher. For example, we found that, among those who reported cigarette use, individuals with a history of criminal justice involvement were younger at initiation, used more cigarettes per day, and were 31% more likely to screen positive for nicotine dependence. Earlier age of initiation and higher rates of dependence likely explain the significantly higher rates of COPD we found among individuals who used cigarettes with a history of criminal justice involvement compared to those with no criminal justice involvement. There were no significant differences in the rate of heart conditions among individuals with and without criminal justice involvement, although the available question within NSDUH does not specifically refer to heart conditions known to be strongly associated with smoking, for example, coronary artery disease [32]. Both the higher prevalence and intensity of tobacco use among individuals likely mediate the relationship between criminal justice involvement and high cardiovascular morbidity and mortality [33].

The United States Preventive Services Task Force recommends that clinicians ask individuals about their tobacco use and, for those that report tobacco use, advise them to quit and offer approved behavioral and pharmacologic interventions [24]. Individuals with criminal justice involvement in this study were less likely to report any outpatient visits, and thus, had fewer opportunities for guideline-based counseling. Among individuals with at least one outpatient visit in the past year, those with criminal justice involvement were less likely to report having been asked about their tobacco use compared to individuals with no criminal justice involvement. However, after adjusting for sociodemographic differences, individuals with criminal justice involvement were more likely to report being advised to quit using tobacco, largely due to the moderating effect of age. This discrepancy may also be related to heavier use among individuals with criminal justice involvement—physicians are more likely to advise heavy smokers to quit [34].

Several opportunities exist to reduce the high burden of tobacco use among individuals with a history of criminal justice involvement. First, clinicians should ask all individuals who are either on community supervision or have been recently incarcerated about their tobacco use and advise them to quit [30]. Second, programs that provide tobacco cessation resources post-release from prison have shown to be modestly effective in improving abstinence after release from prisons with smoking bans [11]. However, the majority of individuals involved in the criminal justice system either spend time in county jail, on community supervision, or both. Smoking cessation programs for individuals on community supervision have had null findings [13], but have potential to reach the largest population of justice-involved individuals. Future work should target the immediate period post-release from jail as an optimal period in which to target tobacco abstinence. Because many jails are now smoke-free, individuals would have a period of forced abstinence to build upon. Such an approach could facilitate cessation among most individuals who are briefly incarcerated prior to a community supervision sentence, those who are revoked while on community supervision, and individuals with sentenced jail stays.

There are important limitations to consider when interpreting the results of this study. The NSDUH is a cross-sectional survey of the US population. As such, we cannot comment on the causal direction of the association between tobacco use and criminal justice involvement. All outcomes are self-reported in the NSDUH and, while anonymous, may still be prone to response bias. Our estimates of tobacco use among justice-involved individuals are likely conservative, because those who are most vulnerable, individuals who are currently incarcerated or homeless, are not included in the NSDUH. Finally, measures of tobacco treatment (e.g., nicotine replacement therapy or pharmacologic therapy) or electronic cigarette use are not available in the NSDUH.

## Conclusions

Tobacco use prevalence among individuals involved in the criminal justice system is more than double the prevalence of tobacco use in the general population, and disparities have worsened over the past 9 years. Novel programs and tobacco control policies are needed to address persistently high rates of tobacco use and thereby reduce cardiovascular morbidity and mortality among individuals with a history of criminal justice involvement.

## Abbreviations

NSDUH: National Survey on Drug Use and Health
COPD: chronic obstructive pulmonary disease
ARD: adjusted relative difference
